# Pharmacological inhibition of human EZH2 can influence a regenerative β-like cell capacity with in vitro insulin release in pancreatic ductal cells

**DOI:** 10.1186/s13148-023-01491-z

**Published:** 2023-06-12

**Authors:** Safiya Naina Marikar, Keith Al-Hasani, Ishant Khurana, Harikrishnan Kaipananickal, Jun Okabe, Scott Maxwell, Assam El-Osta

**Affiliations:** 1 Present Address: Epigenetics in Human Health and Disease Program, Baker Heart and Diabetes Institute, 75 Commercial Road, VIC 3004 Melbourne, Australia; 2 Department of Diabetes, Central Clinical School, Monash University, VIC 3004 Melbourne, Australia; 3 Epigenetics in Human Health and Disease Laboratory, Central Clinical School, Monash University, Melbourne, VIC 3004 Australia; 4 Department of Medicine and Therapeutics, The Chinese University of Hong Kong, Sha Tin, Hong Kong SAR; 5 Hong Kong Institute of Diabetes and Obesity, Prince of Wales Hospital, The Chinese University of Hong Kong, 3/F Lui Che Woo Clinical Sciences Building, 30‑32 Ngan Shing Street, Sha Tin, Hong Kong SAR; 6 Li Ka Shing Institute of Health Sciences, The Chinese University of Hong Kong, Sha Tin, Hong Kong SAR; 7 Biomedical Laboratory Science, Department of Technology, Faculty of Health, University College Copenhagen, Copenhagen, Denmark

**Keywords:** Islet, Pancreas, Ductal cells, Regenerative capacity, Histone modification, EZH2

## Abstract

**Background:**

Therapeutic replacement of pancreatic endocrine β-cells is key to improving hyperglycaemia caused by insulin-dependent diabetes . Whilst the pool of ductal progenitors, which give rise to the endocrine cells, are active during development, neogenesis of islets is repressed in the human adult. Recent human donor studies have demonstrated the role of EZH2 inhibition in surgically isolated exocrine cells showing reactivation of insulin expression and the influence on the H3K27me3 barrier to β-cell regeneration. However, those studies fall short on defining the cell type active in transcriptional reactivation events. This study examines the role of the regenerative capacity of human pancreatic ductal cells when stimulated with pharmacological inhibitors of the EZH2 methyltransferase.

**Results:**

Human pancreatic ductal epithelial cells were stimulated with the EZH2 inhibitors GSK-126, EPZ6438, and triptolide using a 2- and 7-day protocol to determine their influence on the expression of core endocrine development marker *NGN3*, as well as β-cell markers insulin, *MAFA*, and *PDX1*. Chromatin immunoprecipitation studies show a close correspondence of pharmacological EZH2 inhibition with reduced H3K27me3 content of the core genes, *NGN3*, *MAFA* and *PDX1*. Consistent with the reduction of H3K27me3 by pharmacological inhibition of EZH2, we observe measurable immunofluorescence staining of insulin protein and glucose-sensitive insulin response.

**Conclusion:**

The results of this study serve as a proof of concept for a probable source of β-cell induction from pancreatic ductal cells that are capable of influencing insulin expression. Whilst pharmacological inhibition of EZH2 can stimulate secretion of detectable insulin from ductal progenitor cells, further studies are required to address mechanism and the identity of ductal progenitor cell targets to improve likely methods designed to reduce the burden of insulin-dependent diabetes.

**Supplementary Information:**

The online version contains supplementary material available at 10.1186/s13148-023-01491-z.

## Introduction

Among the top 10 leading causes of deaths worldwide, treatment of diabetes remains problematic due to a lack of curative therapeutics, with vigorous vigilance required to maintain normoglycaemia in patients. Whilst transplantation of whole pancreas or purified islets is effective at restoring the glucose index in Type 1 diabetics [1] with the condition, this is severely limited by the numbers of donors worldwide. Given the projected increase in prevalence of diabetes [2], replacement of the insulin-producing β-cells remains an unmet medical need.

**Fig. 1:**
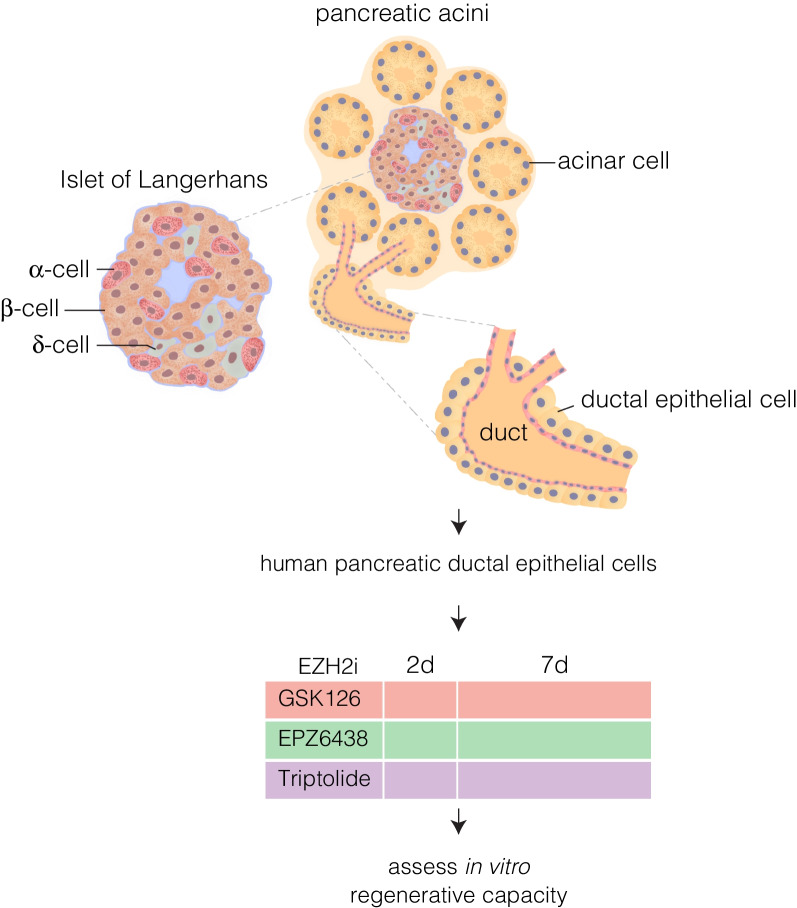
Schematic organization of the exocrine and endocrine compartments in the human pancreas featuring the ductal epithelial cells which are hypothesized to contain progenitor cells capable of regeneration upon exposure to EZH2 inhibitors (EZH2i)

Pancreas organogenesis is tightly regulated by the stepwise expression of transcription factors (TFs) that generates both the exocrine (consisting of the acinar and ductal cells), and endocrine (Islets of Langerhans) compartments (Fig. 1) [3–10]. The existence of stem cells within the pancreatic ducts has been widely debated with observational studies depicting the aggregation of islets adjacent to the ductal epithelium suggestive of their ductal origin [11–14]. Further pancreatic injury models, ranging from pancreatic ductal ligation to partial and 90% pancreatomies, offered support for the hypothesis that stem cells were present within the ductal niche [15] and could give rise to endocrine cells of the islet upon *NGN3* expression, similar to embryonic development [16]. More recent studies have demonstrated the existence of ductal *NGN3*+ cells with the ability to differentiate and give rise to adult β-cells [17]. The results of that study were correlated by single cell RNA sequencing experiments of the ductal progenitor niche [18] and reconfirms evidence from a prior study which demonstrate the presence of ductal progenitors capable of differentiating into α-cells, followed by development into β-cells upon overexpression of Pax4, or inhibition of Arx [19, 20]. Lineage tracing studies of this process clearly demonstrate the existence of ductal *NGN3*+ cells that have differentiated into insulin-producing β-cells. Although highly active during development, the mechanism of de novo generation of the endocrine compartment by ductal progenitor cells is suppressed in the adult [21, 22], whereas the exocrine compartment is thought to be considerably more plastic. This raises questions to the mechanisms by which this repression is achieved, as well as how it may be reactivated to restore the β-cell mass.

A recent study provided evidence for the role of epigenetics in the repression of pancreatic progenitors with the presence of epigenetic modifications differing between ductal progenitors capable of differentiating into β-cells via transgenic overexpression of *PAX4* or inhibition of *ARX*, and the latent progenitor population of wild-type controls [23]. The inhibition of *NGN3*-positive progenitors is associated with the alteration of histone modifications that are responsible for functionally repressing genes. Differential EZH2 expression during embryogenesis plays an important role on influencing the generation of endocrine progenitors [24]. Furthermore, EZH2 has been shown to regulate the differentiation of pancreatic endocrine progenitors in the embryonic stage [25] along with playing key roles in other models of disease [26, 27]. Therefore, it may have a function in the repression of ductal progenitors in the adult, with inhibition of the EZH2 protein allowing for activation of the adult ductal progenitors. Indeed, further studies show EZH2 inhibition in a mixed exocrine milieu isolated from human donors, stimulated the production of insulin transcripts, resulting in a change in the expression profile [28]. Given the results of previous studies, we had proposed that the increase in insulin as a result of EZH2 inhibition could originate from the ductal progenitors within the mixed exocrine fraction [28]. To investigate this, human pancreatic ductal epithelial cells were stimulated with EZH2 inhibitors (Fig. 1).

## Results

### Increase in β-cell markers following inhibition of EZH2

**Fig. 2:**
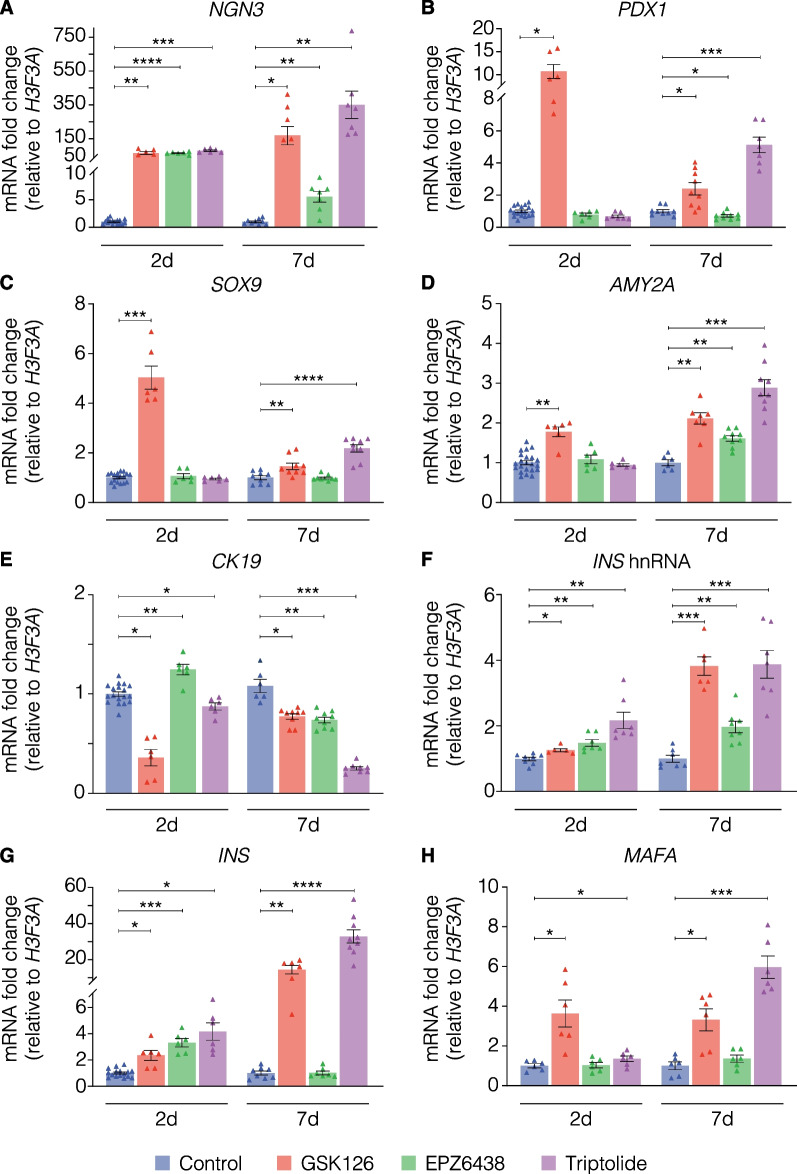
Key markers of endocrine development, epithelial-to-mesenchymal transition, pancreatic ducts, and islets are altered in human pancreatic ductal cells following stimulation with EZH2 inhibitors. Variation in mRNA transcripts of **A** *Neurogenin3* [*NGN3*], **B** *Pancreatic duodenal homeobox factor 1* [*PDX1*], **C** *SRY-box transcription factor 9* [*SOX9*], **D** *Alpha amylase* [*AMY2A*], **E** *Cytokeratin 19* [*CK19*], **F** *Insulin* heterogeneous nuclear RNA [*INS* hnRNA], **G** *Insulin* [*INS*] and **H** *V-maf musculoaponeurotic fibrosarcoma oncogene homolog A* [*MAFA*], following 2- and 7-day stimulation with GSK-126 at 10 µM, EPZ6438 at 1 µM, and triptolide at 20 nM. Data are displayed as mean of fold change ± S.E.M. of 3 replicates, calculated by normalizing drug values to DMSO (vehicle-treated) controls. Statistically significant change in expression was determined using Student’s t test to compare control values to each drug, *P < 0.05, **P < 0.01, ***P < 0.001, ****P < 0.0001

To examine whether pharmacological EZH2 inhibition could influence the terminal differentiated status, adult human pancreatic ductal epithelial cells were stimulated with GSK-126, EPZ6438 and triptolide over 2 and 7 days (Fig. 2). Genes investigated included those important during endocrine development, as well as the expression of genes involved in retention of identity post-development for both ductal and β-cells. Culture of pancreatic ductal cells with GSK-126, EPZ6438 and triptolide for 2 days significantly elevated *NGN3* mRNA levels (Fig. 2A). Further increases were observed at 7 days with GSK-126 and triptolide. In contrast, only GSK-126 demonstrated a statistically significant increase in *PDX1* expression at 2 days and triptolide demonstrating increase at 7 days (Fig. 2B). A similar observation was seen with *SOX9* expression (Fig. 2C). Alpha-amylase (*AMY2A*) transcripts were increased significantly following 2 days of GSK-126 stimulation, while all EZH2 inhibitors significantly elevated *AMY2A* mRNA levels (Fig. 2D). There was a decrease in the cytoplasmic *CK19* following 2 days of treatment (Fig. 2E) showing statistically significant decreases upon stimulation with GSK-126 and triptolide. Although EPZ-6438 demonstrated an increase at the 2-day time point, by day 7 *CK19* expression levels had decreased significantly to match the reduction seen with the other compounds (Fig. 2E).

Inhibition of EZH2 in pancreatic ductal cells stimulated a statistically significant increase in insulin transcripts of both the unprocessed heterogeneous nuclear form (Fig. 2F) and the mature mRNA (Fig. 2G). The increase in gene expression was maintained at 7 days with even further increases for triptolide, as well as GSK-126. V-maf musculoaponeurotic fibrosarcoma oncogene homolog A, or *MAFA* mRNA levels increased in a statistically significant manner at the 2-day point only upon treatment with GSK-126 and triptolide (Fig. 2H). Whilst the fold change compared to vehicle treatment was stable for GSK-126 treatment at 7 days, EPZ-6438 similarly began to induce a statistically significant increase in *MAFA* transcript.

### H3K27me3 content is reduced in endocrine genes following EZH2 inhibition

**Fig. 3:**
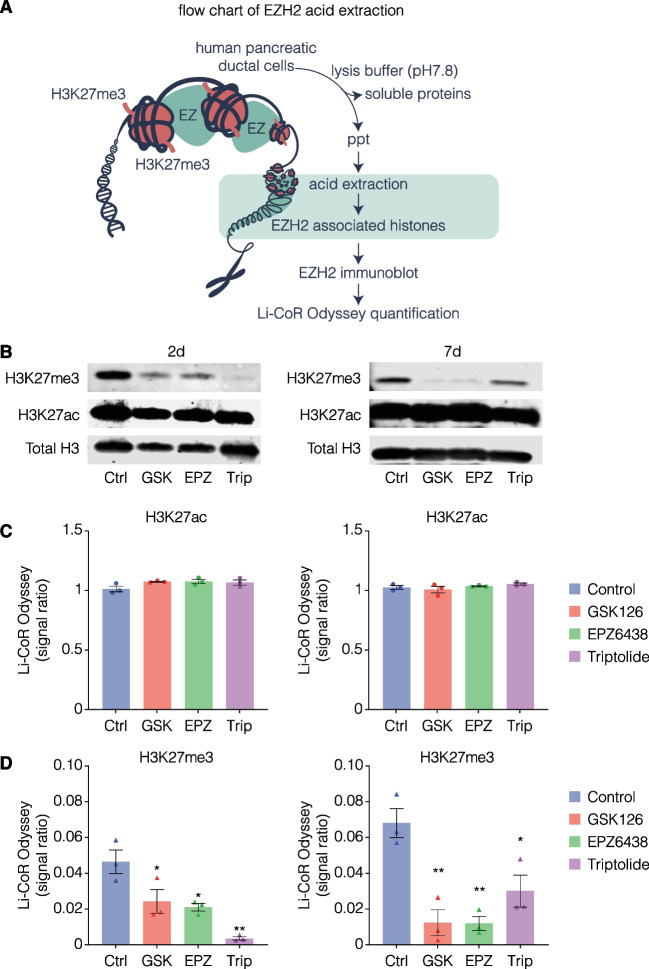
Stimulation with EZH2 inhibitors reduces H3K27me3 content in human pancreatic ductal epithelial cells. **A** Histone proteins were isolated from pancreatic ductal cells stimulated with EZH2 inhibitors and control cells using 5 M of sulfuric acid. Acid-precipitated (ppt) histone proteins were separated on Nu-Page gel followed by immunoblotting to quantify the total H3 and H2K27me3 levels using Li-CoR Odyssey. **B** Representative western blots of H3K27me3 and H3K27ac relative to total H3 following 2-day and 7-day stimulation with GSK126 at 10 µM, EPZ6438 at 1 µM, triptolide at 20 nM compared with vehicle control DMSO. **C** Quantitative analysis of H3K27ac and **D** H3K27me3 relative to total H3 following 2-day and 7-day stimulation with GSK126 at 10 µM, EPZ6438 at 1 µM, triptolide at 20 nM compared with vehicle control DMSO. Data are displayed as mean signal ratio of H3K27ac or H3K27me3 to total H3 ± SEM of 3 replicates with representative blots above. Each dot plot represents signal ratio of H3K27ac from one independent replicate. Each triangle plot represents signal ratio of H3K27ac or H3K27me3 from one independent replicate. Statistically significant differences were determined using Student’s t-tests against control. *P < 0.05, **P < 0.01

To confirm that the EZH2 inhibitors were working to reduce EZH2 activity within the nuclear chromatin, histone proteins were isolated by acid-extraction from pancreatic ductal cells and analysed for H3K27me3 and H3K27ac content relative to total H3 extracted (Fig. 3A). There was a lack of change in H3K27ac between EZH2*i* treatments and controls (Fig. 3 B, C). In contrast, following 2 days, the drugs significantly reduced H3K27me3 in the pancreatic ductal cells when compared to the vehicle-treated controls (Fig. 3B, D). Prolonged stimulation over 7 days further decreased H3K27me3 with GSK126 and EPZ6438, which was not noted with triptolide (Fig. 3D).

**Fig. 4:**
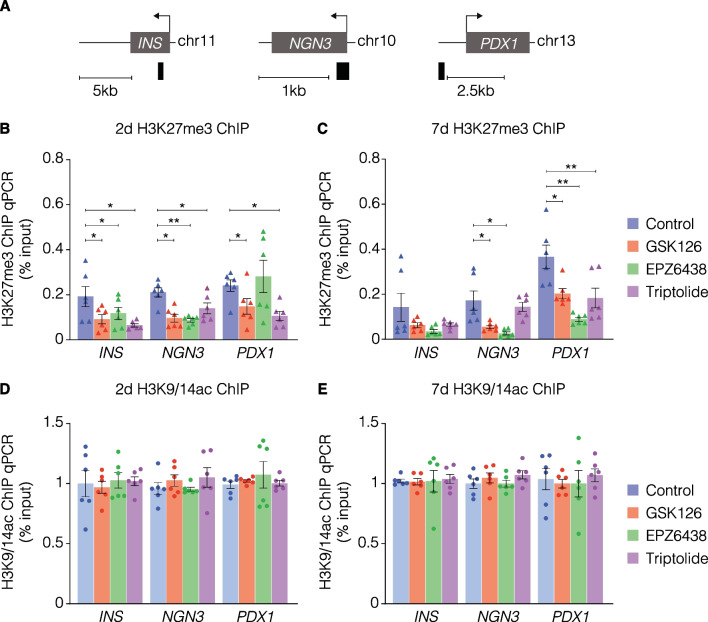
Reduction of H3K27me3 content associated with the chromatin of DNA in the *INS-IGF2*, *NGN3*, and *PDX1* promoter regions following inhibition of EZH2. **A** H3K27me3 content was assessed by using amplifiers (black bars against DNA regions corresponding to the promoters of *INS*, *NGN3* and *PDX1*). Quantitative PCR analysis of H3K27me3 associated DNA using ChIP following **B** 2-day and **C** 7-day stimulation of human pancreatic ductal epithelial cells compared to vehicle control. Data are displayed as the mean input signal against H3K27me3 abundance ± S.E.M of 3 replicates. Each triangle plot represents one technical replicate. Statistically significant differences were determined using Student’s *t*-tests against control. *P < 0.05, **P < 0.01, ***P < 0.001. Quantitative PCR analysis of H3K9/14ac associated DNA using ChIP following **D** 2-day and **E** 7-day stimulation of human pancreatic ductal epithelial cells compared to vehicle control. Data are displayed as the mean input signal against H3K9/14ac abundance ± S.E.M of 3 replicates. Each dot plot represents one technical replicate. Statistically significant differences were determined using Student’s t-tests against control

Given that EZH2 is involved in writing H3K27me3 which is associated with gene repression, we next assessed whether gene expression as a result of treatment with EZH2 inhibitors could influence H3K27me3 content of related genes. H3K27me3-associated chromatin was immunopurified (ChIP) from pancreatic ductal cells. qPCR was used to assess H3K27me3 content using primers specifically designed to detect the promoter regions of *INS*, endocrine master regulator *NGN3*, and β-cell marker *PDX1* (Fig. 4A). Consistent with previous results [28], ChIP revealed GSK-126 significantly reduced the H3K27me3 content of chromatin associated with the *INS* promoter domain, as well as *NGN3* and *PDX1* (Fig. 4B). Additionally, these results were also demonstrated with triptolide. EPZ-6438 reduced H3K27me3 content of *PDX1* at 7 days (Fig. 4C). Furthermore, a lack of change in the H3K9/14 acetylation (H3K9/14ac) demonstrates the specificity of the EZH2*i* in modulating the trimethylation signal of human pancreatic ductal epithelial cells (Fig. 4D, E), which is further correlated with a lack of change as assessed by western blots (Additional file 1: Figure S1).

### EZH2 inhibition stimulates insulin expression in human pancreatic ductal epithelial cells

**Fig. 5:**
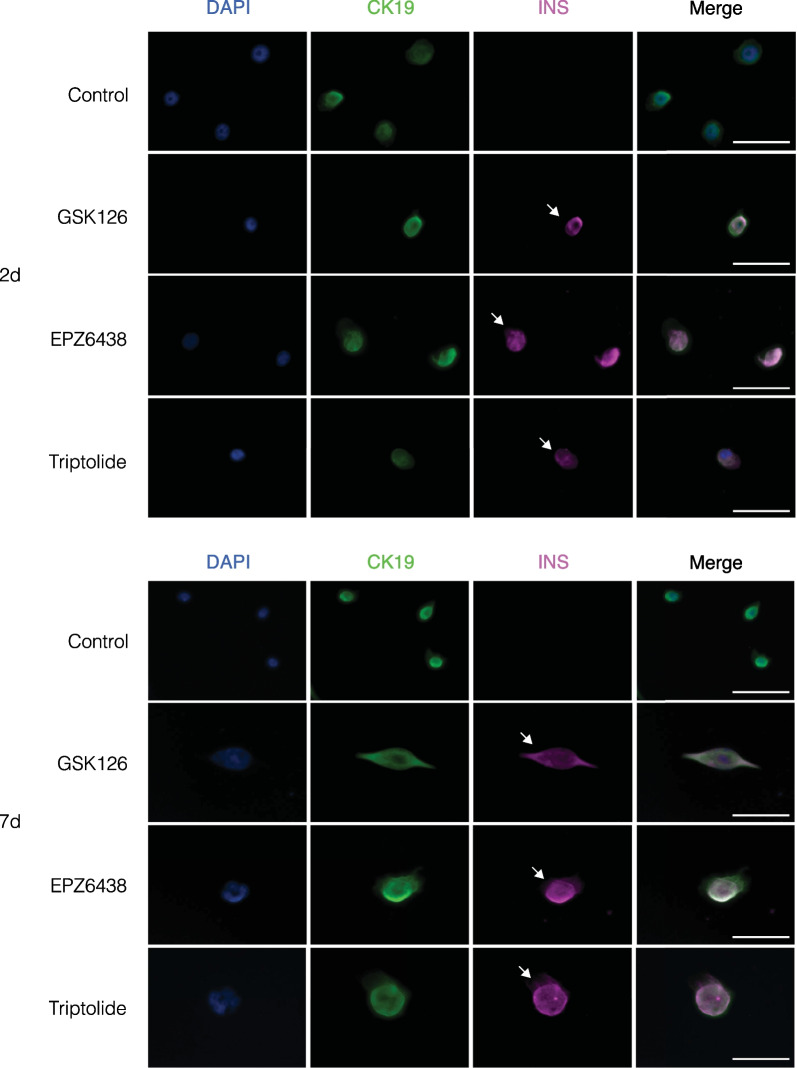
Stimulation of human pancreatic ductal cells with EZH2 inhibitors GSK-126 at 10 µM, EPZ6438 at 1 µM and triptolide at 20 nM influences the expression of insulin (*INS*) following **A** 2-day and **B** 7-day stimulation. Images were captured using ThermoFisher EVOS at 40×magnification and processed on ImageJ. Images are representative of 3 replicates. Scale bar represents 50 µM. White arrows indicate *INS* expressing *CK19*-positive cells

To determine whether increased *INS* and *CK19* gene expression were indicative of a functional synthesis of the protein, cells stimulated with EZH2 inhibitors for 2 and 7 days were stained using immunofluorescence for INS and CK19 with DAPI serving as a control nuclear stain (Fig. 5A). All cells stained were positive for CK19 indicating their ductal cell identity. Importantly, a population of pancreatic ductal cells were positive for insulin, which were not present in the DMSO controls. An average of 3 in 20,000 cells were observed. Following 7 days, there was an overall increase in the numbers of insulin-positive cells, averaging 7 in 20,000 cells per treatment (Fig. 5B).

### Human pancreatic ductal epithelial cells are capable of releasing insulin

**Fig. 6:**
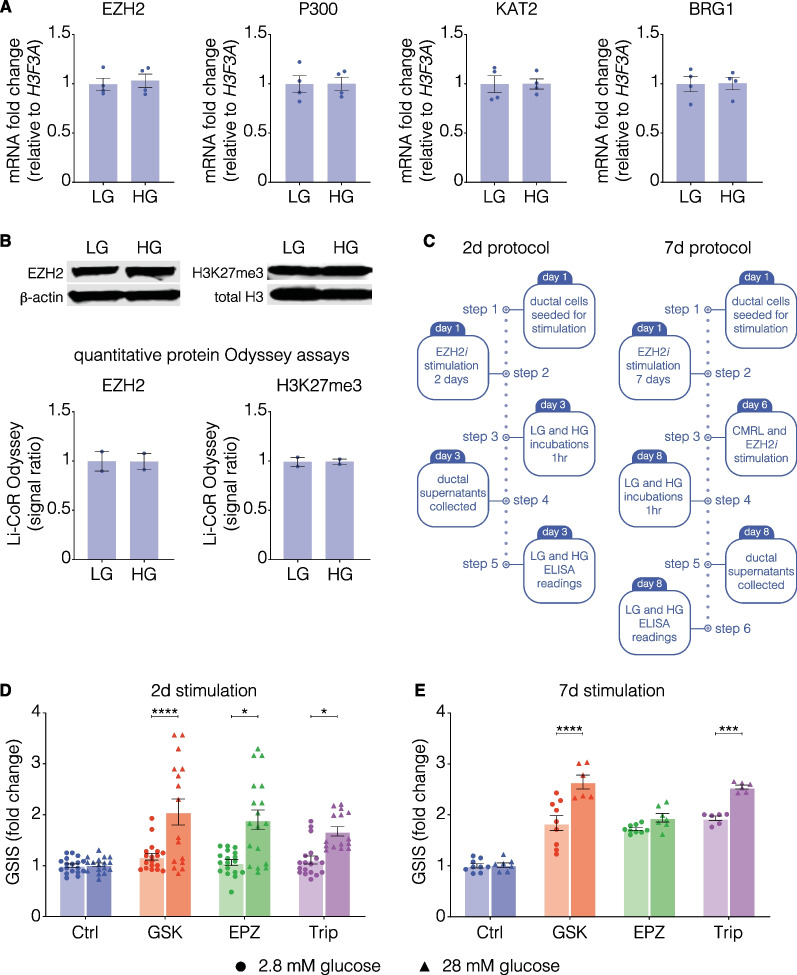
EZH2 inhibitors influence glucose-sensitive insulin secretion in human pancreatic ductal cells. **A** Gene expression of chromatin modulators EZH2, P300, KAT2 and BRG1 are unchanged in non-inhibitor-treated cells following exposure to high glucose (HG) compared to non-exposed (LG) cells. Data are presented as mean of fold change ± S.E.M. of 2 replicates, calculated by normalizing high glucose values to low glucose (unexposed) controls. Statistically significant change in expression was determined using Student’s *t*-test. **B** Representative western blots and quantitative analysis of EZH2 and H3K27me3 following exposure to high glucose (HG) compared to low glucose (LG). Data are displayed as mean signal ratio of EZH2 to β-actin or H3K27me3 to total H3 ± SEM of 2 replicates with representative blots above. Each dot plot represents signal ratio of one independent replicate. Statistically significant differences were determined using Student’s *t*-tests against control. **C** 2- and 7-day protocols for assessment of glucose-stimulated insulin secretion from EZH2 inhibitor (EZH2*i*) treated human pancreatic ductal epithelial cells. Both protocols were initiated with seeding of cells to establish cultures. Two-day EZH2*i* stimulation was performed in CMRL to resolve background insulin, whilst for 7-day stimulations, the initial EZH2*i* doses were delivered in normal growth media, followed by switching to CMRL on day 6. On the final day of the protocol, cells were incubated for 1 h in low glucose followed by 1 h in high glucose. The supernatant was collected for quantification of insulin secretion in ELISAs. ELISA quantified **D** 2- and **E** 7-day secretion of insulin from human pancreatic ductal epithelial cells following 1 h of incubation in low (2.8 mM) and high (28 mM) concentrations of glucose. Insulin concentrations were normalized to control 2.8 mM concentrations to calculate fold change. Data are presented as mean of fold change ± S.E.M. of 3 replicates. Dots represent one technical replicate of 2.8 mM glucose supernatant. Triangles represent one technical replicate of 28 mM glucose supernatant. Student’s *t*-tests were used to assess whether variation in insulin secretion was statistically significant, *P < 0.05, **P < 0.01, ****P < 0.0001

Since insulin was detected by pharmacological EZH2 inhibition, we assessed glucose-stimulated insulin secretion (GSIS). To confirm the specificity of the assay, basal and stimulating levels of EZH2 and H3K27me3 were established by qRT-PCR (Fig. 6A), as well as western blot (Fig. 6B). Additionally, mRNA levels of the histone acetyltransferases P300, KAT2, and chromatin remodeler BRG1 were unchanged following 1 h of incubation in high glucose. There was no change in EZH2 expression, or protein levels as demonstrated by the lack of change in trimethylation following exposure to high glucose, thus confirming that any subsequent alterations in insulin concentration were due to EZH2*i* stimulation. The GSIS assay was performed following EZH2*i* stimulation using a 2 or 7 day protocol (Fig. 6C) to examine whether those insulin-producing pancreatic ductal cells were functional in their capacity to produce insulin under 1 h of incubation in basal (2.8 mM glucose) or stimulating (28 mM glucose) conditions. Following incubation in high-glucose media, we observed increased release of insulin from human pancreatic ductal epithelial cells stimulated with GSK-126, EPZ6438 and triptolide (Fig. 6D), which was not observed in the control DMSO-treated cells. Release of insulin was also maintained at 7 days with GSK126 and triptolide (Fig. 6E). Taken together these results suggest there is a population of cells capable of releasing insulin following pharmacological EZH2 inhibition.

## Discussion

**Fig. 7:**
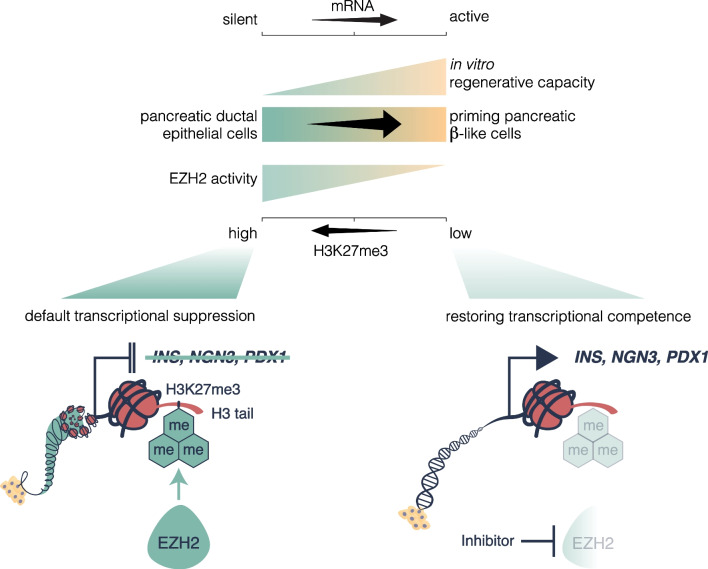
The default transcriptional state of pancreatic ductal progenitors is hypothesized by high EZH2 activity suppressing the expression of endocrine genes such as *INS*, *NGN3* and *PDX1*. Inhibition of EZH2 restores the progenitor capacity in ductal cells, reducing the H3K27me3 mark and allowing for transcription of genes that influence differentiation into β-like cells

While the default regenerative state of ductal cells is thought to lie dormant in the adult pancreas, the experimental observations of these studies indicate that adult pancreatic ductal progenitor cells can be influenced and re-activated upon stimulation by EZH2 inhibition (Fig. 7).

EZH2 is a known epigenetic regulator capable of binding to the master controller of pancreatic endocrine cells *NGN3* and considered an important regulator of endocrine fate during development [29]. Consistent with previous studies demonstrating the ability of ductal cells to transdifferentiate from α-cells into insulin-producing cells [19, 20], we observe that the transcriptional expression index of human pancreatic ductal epithelial cells stimulated by pharmacological EZH2 inhibitors can be recapitulated in terminally differentiated adult cells, with increased expression in *NGN3* indicating a transition in cell identity resulting from exposure to GSK-126, EPZ-6438, and triptolide. This result closely corresponds with the reduced H3K27me3 chromatin content associated with the *NGN3* gene, implying a function for EZH2 in supressing the progenitor population found in the adult pancreatic ductal niche. Indeed, the loss of the repressive H3K27me3 mark plays a role in allowing for the activation of developmental regulators in a number of systems including pancreatic and endocrine formation [30–32]. These developmental regulators, including *PDX1*, are known to be bivalently primed, possessing both the repressive H3K27me3 and activating H3K4me3 mark, with EZH2 as part of the PcG group playing an important role in maintaining repression in non-endocrine cell types, as well as in endocrine progenitors. *SOX9* is considered the master regulator of epithelial-to-mesenchymal transition in a number of developmental processes [5, 7] responsible for regulating downstream genes involved in proliferation and differentiation. The increase in *SOX9* gene expression probably reflects the developmental recapitulation of epithelial-to-mesenchymal transition following *NGN3* expression as the ductal pancreatic progenitors aggregate to form the endocrine islets, a process that is essential for islet cells to maintain their function by defining their structure [33]. Decreased *CK19* expression further reflect this transition, by indicating a subtle yet functionally important loss of ductal cell identity.

Despite elevated *NGN3* expression following 2 days, only GSK-126 resulted in a significant tenfold increase of *PDX1* transcripts, with longer periods required to stimulate similar increases in *PDX1* by other compounds. These results may indicate a greater efficacy of GSK-126 over an identical time period, with more delayed effects demonstrated by the other drugs due to structural variations affecting binding efficacy. Although responsible for inducing pancreatic formation during embryogenesis [34] *PDX1* expression in the adult is confined to the β-cell and is critically involved in regulating insulin production in the mature β-cell. Strikingly, 2 days of stimulation was sufficient to demonstrate increased insulin gene expression, as well as protein, albeit in a select number of cells. Interestingly, the elevation of *INS* hnRNA implies a functional de novo transcription of the insulin gene, which closely correlates with the expression pattern of the mature *INS* mRNA, [35] further corroborating evidence of the increase in insulin gene expression. In addition, although *CK19* gene expression was reduced when treated with GSK-126 at 2 days, with EPZ-6438 and triptolide reducing expression at 7 days, immunofluorescent staining demonstrated no overt reduction in the protein level, possibly indicating a retention of ductal phenotype and representing a more transitionary immature β-cell albeit increased expression of *MAFA*, which is restricted to mature β-cells in adults similar to *PDX1* [36]. Indeed, previous studies have further noted heterogeneity among juvenile and transitionary β-cells, which may reflect the co-staining of insulin and *CK19* in this cell population [37]. Importantly, *MAFA* is critically involved in regulating the ability of β-cells to respond to glucose [38], which may explain the sensitivity to glucose variability. Furthermore, the results of the GSIS assay highlight an important feature of epigenetically reprogrammed insulin-producing cells, namely their ability to reflect mature β-cells by responding to changes in glucose levels by secreting insulin. It is important to note, however, that the increase in the concentration of insulin is at a picomolar level, with mature β-cells demonstrating variations with much larger increases of concentration [39]. Indeed, although staining of 7-day cells displayed a consistent *CK19* signal, the increase in insulin positive cell size was more indicative of a progression of the differentiation process, with elevated basal insulin secretion more accurately reflecting physiological conditions displayed by β-cells [39].

As potential therapeutics, GSK-126, and triptolide appear to exert pharmacological effects on transcriptional indices associated with β-cell neogenesis and identity, with GSK-126 showing a greater efficacy at day 2 and triptolide at day 7, displaying an over 300-fold increase in gene transcription in the case of *NGN3*. This disparity in the transcriptional expression index may lie in the differing modes of action for GSK-126 [40], which is a synthetically designed competitive inhibitor of EZH2, whilst triptolide —a naturally derived EZH2 inhibitor that has been shown to reduce EZH2 at the protein level by inhibiting EZH2 gene transcription [41]. Whilst GSK-126 usage has been primarily trialled as a cancer therapeutic, triptolide has been tested as a treatment for inflammatory conditions [42]. However, there was no measurable difference between the three EZH2 inhibitors on insulin secretion, correlating with their similar effect on the nuclear H3K27me3 content of adult pancreatic ductal cells. Additionally, although GSK-126 and EPZ6438 are both S-adenosyl-l-methionine (SAM) competitive inhibitors of EZH2, they display varied effects on differential gene expression. This might be due to the aforementioned variation in binding capacity, with GSK-126 demonstrating greater specificity to EZH2 relative to EZH1 when compared to EPZ6438 [43]. The specific effects of EZH2 inhibition will need to be explored in subsequent knockdown or silencing experiments to conclusively determine the differential expression profile induced by inhibition of EZH2 unobscured by any off-target effects arising due to the lack of drug clearance in vitro.

This work, albeit important as a proof-of-concept study but also recognizing its limitations, will require evaluation in a suitable diabetic animal model to determine whether the functional response to glucose by inhibiting pancreatic EZH2 is capable of ameliorating hyperglycaemia through a duct-to-β-cell regenerative pathway. The results bear weight given that, although limited in their ability to model normal physiology, the ductal cell line use reflects human epigenetic modifications, which are not always recapitulated in murine models with noted variations between species extending from genetic and cellular regulation to tissue structure as well as overall function [39, 44, 45].

Importantly, based on the results of immunofluorescent staining for insulin, not all ductal cells are capable of responding to EZH2 inhibitor stimulation, highlighting the as-of-yet undefined pool of ductal progenitors that can express *NGN3* and differentiate into insulin-producing cells. The differential cellular response reflects the diminutive numbers of progenitor cells, which have been noted previously, as well as reports of heterogeneity in pancreatic cell populations both at the embryonic and the adult stage using single cell analysis [18, 46, 47]. Possible approaches to better characterize these progenitor populations may involve single-cell sequencing to examine which genes or markers can be used to define cells capable of undergoing differentiation into β-like cells. Furthermore, whilst insulin protein expression was detected in our studies, additional experiments need to be conducted to confirm the cellular presence of the other endocrine markers to determine whether these ductal progenitors are able to generate other islet cell types. Likewise, although the inhibition of EZH2 is shown to reduce the H3K27me3 mark, allowing for *NGN3* transcription, the specific genes that are differentially regulated downstream of this process and responsible for this transition are yet to be explored.

In summary, while novel therapies that restore β-cell mass are required to effectively ameliorate deficiencies associated with insulin-dependent diabetes, our study serves as a proof of concept that EZH2 inhibition could influence reprogramming of adult human pancreatic ductal cells towards insulin expressing and glucose responsive β-like transitioning cells following 48 h of exposure to inhibitors. Whilst extensively covered during development [25, 30], this is the first paper to determine a role for EZH2 in endocrine cell determination, as well as maintenance of pancreatic ductal progenitors in the adult. Our experimental observations suggest the possibility that reprogrammed cells were capable of producing insulin and functionally elevated insulin secretion in response to glucose stimulation. While the study was limited to in vitro demonstration of EZH2 inhibition, future studies will need to account for heterogeneity in cell populations, as well as investigating the ability to reverse hyperglycaemia in pre-clinical models whilst further characterizing the responsive-regenerative cells. In conclusion, this study provides some evidence that there exists a niche of pancreatic ductal cells that are capable of becoming β-like cells and therefore representing a viable alternative source for cell replacement therapy.

## Methods

### Cell culture and EZH2 inhibitor stimulation

Human pancreatic ductal epithelial cells were purchased from AddexBio and cultured according to the recommended protocols. Cells were cultured in complete Keratinocyte Serum-Free Media (supplemented with human recombinant EGF, Bovine Pituitary Extract and Antibiotic–Antimycotic [Gibco]). All cell cultures were grown and maintained in a 37 °C, 5% CO2 environment using a tissue culture incubator. Once cells reached 70–80% confluency, passaging was performed using 0.05% Trypsin EDTA (Sigma).

EZH2 inhibitors investigated in this study included the synthetically designed GSK-126 (S7061, SelleckChem), and EPZ-6438 (S7128, SelleckChem), as well as the naturally occurring compound, triptolide (S3604, SelleckChem), which is known to display EZH2 inhibitor activity. Vehicle control was DMSO. Cells were treated over 2 main timepoints, with harvests occurring following 2 and 7 days.

For the 2-day time point, cells were seeded and left to adhere in plates for 24 h. Treatment was initiated with the first dose made up in complete K-SFM. The second dose was delivered 24 h later following a media change. For the longer period of 7 days, cells were initially seeded in 10-cm plates, with doses delivered on alternate days following the initial period of 24 h. When the plate reached 90% confluency, cells were passaged using 0.05% trypsin EDTA (Sigma) and re-seeded at a 1:2 dilution. 3 days prior to harvest, cells were passaged and seeded into cell culture plates depending on the application, with the final addition occurring like the 2-day treatment over 2 periods of 24 h.

### RNA extraction and quantitative RT-PCR

TRIzol was used to extract total RNA from 5 × 10^5^ cells seeded in 12-well plates, which were untreated (vehicle control DMSO) or incubated with EZH2 inhibitors for 2 and 7 days. RNA was isolated using the RNeasy Kit according to the manufacturer’s directions. Following measurement of RNA concentration using a QIAxpert System, 1 ug of RNA was used for cDNA synthesis by a high-capacity cDNA Reverse Transcription Kit (Applied Biosystems). The resulting cDNA reaction mix was diluted 1:6 to make up the final template cDNA used subsequently.

Quantitative real-time PCR (qRT-PCR) was performed to examine differential gene expression using the following reaction mix: 5 µL Brilliant II SYBR^®^ Green QPCR Master Mix (600,903, Agilent Technologies), 2 µL nuclease-free water, 2 µL of template cDNA, and 0.5 µL of forward and reverse primer from OligoPerfect designer (Thermo Fisher Scientific), detailed in Table 1. qRT-PCR cycles were carried out using Applied Biosystems 7500 Fast Real-Time PCR System, with each reaction consisting of a 3-min hot start at 95 °C, followed by 40 cycles of 5 s at 95 °C, and 15 s at 60 °C. Ct values of experimental genes were normalized to housekeeping gene H3F3A. Fold change of mRNA abundance was calculated by normalizing drug treated values to vehicular controls.

**Table 1:** Human primers for qRT-PCR

Gene	Primer	Sequence
H3F3A	Human cDNA forward	ACAAAAGCCGCTCGCAAGAGTG
	Human cDNA reverse	TTTCTCGCACCAGACGCTGGAA
INS	Human cDNA forward	GCAGCCTTTGTGAACCAACAC
	Human cDNA reverse	CCCCGCACACTAGGTAGAGA
NGN3	Human cDNA forward	CTAAGAGCGAGTTGGCACTGA
	Human cDNA reverse	GAGGTTGTGCATTCGATTGCG
PDX1	Human cDNA forward	GAAGTCTACCAAAGCTCACGCG
	Human cDNA reverse	GGAACTCCTTCTCCAGCTCTAG
SOX9	Human cDNA forward	AGGAAGCTCGCGGACCAGTAC
	Human cDNA reverse	GGTGGTCCTTCTTGTGCTGCAC
CK19	Human cDNA forward	AGCTAGAGGTGAAGATCCGCGA
	Human cDNA reverse	GCAGGACAA TCCTGGAGTTCTC
AMY2A	Human cDNA forward	GATAATGGGAGCAACCAAGTGGC
	Human cDNA reverse	CAGTATGTGCCAGCAGGAAGAC
MAFA	Human cDNA forward	GCTTCAGCAAGGAGGAGGTCAT
	Human cDNA reverse	TCTGGAGTTGGCACTTCTCGCT
INS hnRNA	Human cDNA forward	GAGATGGGGAAGATGCTGGG
	Human cDNA reverse	GGAGGACACAGTCAGGGAGA
EZH2	Human cDNA forward	TCCTTTTCATGCAACACCCAACACT
	Human cDNA reverse	TCCAAATGCTGGTAACACTGTGGTC
EP300	Human cDNA forward	GCAGTGTGCCAAACCAGATG
	Human cDNA reverse	GGGTTTGCCGGGGTACAATA
KAT2	Human cDNA forward	ATTCTGCAGGGGCCGAGCCT
	Human cDNA reverse	ATCACACGGAGCCGCTTGGC
BRG1	Human cDNA forward	GCTCATGGCTGAAGATGAGG/
	Human cDNA reverse	CAGGCGCTTGTCCTTCTTC

### Quantitative PCR Chromatin immunoprecipitation (q-PCR ChIP)

Approximately 5 × 10^6^ cells were fixed in 1% formaldehyde for 10 min, with a further 10-min incubation in 0.125 M glycine to quench the cross-linking reaction. The fixed cell pellet was lysed following resuspension and homogenization in sodium dodecyl (lauryl) sulphate (SDS) lysis buffer (1% SDS, 10 mM EDTA, 50 mM Tris–HCl pH 8.1) with a protease inhibitor cocktail (Roche Diagnostics GmBH, Mannheim, Germany) included. Samples were incubated on ice for 5 min following which sonication was performed to shear chromatin between 200 and 600 bp. Sonicated chromatin was resuspended in ChIP Dilution Buffer (0.01% SDS, 1.1% Triton X-100, 1.2 mM EDTA, 16.7 mM Tris–HCl pH 8.0 and 167 mM NaCl). 20 μL of Dynabeads^®^ Protein A (Invitrogen, Carlsbad, CA, USA) was added to each sample and pre-cleared. Overnight incubation at 4 °C with H3K27me3 or H3K9/14ac antibody was used for immunoprecipitation of chromatin, as previously described. Immunoprecipitates were collected by magnetic isolation and washed sequentially with low-salt and high-salt buffers. Immunoprecipitated DNA was then eluted from solution with 0.1 M NaHCO3 containing 1% SDS. Protein-DNA cross-links were reversed by incubation of samples in Proteinase K (Sigma, St. Louis, MO, USA) for 2 h at 62 °C. DNA was purified using a Qiagen MinElute column (Qiagen Inc., Valencia, CA, USA). H3K27me3 or H3K9/14ac content at the promoters of the INS-IGF2, NGN3 and PDX1 genes was assessed by qPCR using primers designed from the integrative ENCODE resource. ChIP primers are shown in Table 2.

**Table 2:** Human primers for q-PCR ChIP

Gene	Primer	Sequence
INS-IGF2	PromR1 forward	GGGAACATAGAGAAAGAGGTCTCA
	PromR1 reverse	AATTAATCTCAGCTTCCCCCTAAC
NGN3	PromR1 forward	TTGCTCCTAGCCTATCTTTCCTTA
	PromR1 reverse	CTTTAGAATTCCTGGACCCTTCTC
PDX1	PromR1 forward	ACGTTTCTGCAAAGCTGTCTAGTT
	PromR1 reverse	GGCTTCAAACCATTCAGTAACTTC

### Protein blot

Histone proteins were extracted from 1 × 10^6^ cells per sample. Acid extraction of nuclear proteins and immunoblotting was performed as previously described [26]. Protein content of samples was incubated using Bradford’s Reagent (Sigma), with standard concentrations of BSA used to determine concentration. 1 µg of protein per sample was run on a 4–12% gel (Nu-Page, Invitrogen) before transfer to a PVDF membrane. Membranes (Immobilon-FL; Millipore) were incubated in primary antibody against H3 (1B1B2, CST), H3K27ac (ab4729, Abcam), EZH2 (#4905, CST), β-actin (ab8226, Abcam) and H3K27me3 (07-229, Millipore) overnight (dilutions listed in Table 3). Membranes were incubated in secondary antibody and imaged using LiCoR Odyssey infrared system. Image studio was used to quantify the protein bands with total H3 or β-actin as a loading control.

**Table 3:** Antibody dilutions for western blot and immunofluorescent staining of human pancreatic ductal epithelial cells

Antibody	Dilution
Rabbit H3K27me3	1:2500
Mouse total H3	1:1000
Rabbit H3K27ac	1:1000
Mouse β-actin	1: 10,000
Rabbit EZH2	1:1000
IRDye^®^ 680CW goat anti-rabbit	1:10,000
IRDye^®^ 800CW goat anti-mouse	1:10,000
Rabbit anti-CK19	1:200
Guinea pig anti-insulin	1:250
Alexa fluor 488 donkey anti-rabbit	1:1000
IRDye^®^ 680CW donkey anti-guinea pig	1:1000

### Immunofluorescence

20 × 10^4^ cells were seeded on 15-mm coverslips in 24-well plates and treated with EZH2 inhibitors or vehicle control over 2 or 7 days. Cells were fixed in 4% PFA. 0.1% Triton X diluted in PBS was used to permeabilize cells for 10 min, followed by blocking in a solution of 0.2% gelatin, 2.5% bovine serum albumin made up in PBS (PBG). Primary antibodies against CK19 (HPA002465 Sigma-Aldrich) and INS (A0564, DAKO) were made up in PBG according to the dilutions listed in Table 3 and incubated overnight at 4 °C. Coverslips were washed and incubated with secondary antibodies against rabbit (Alexa Fluor 488), and guinea pig (IRDye^®^ 680CW) (dilutions in Table 3) for 1 h at room temperature. Cells were then washed and incubated with 4′,6-diamidino-2-phenylindole (DAPI) as a nuclear counterstain (at a 1:100 dilution from a 10 μg/mL stock; D8417 Sigma-Aldrich) for 10 min prior to mounting using Prolong Gold Anti-Fade mountant with DAPI (ThermoFisher). Slides were viewed and images were obtained from EVOS (ThermoFisher) using the TagBFP, Cy5, and GFP filters. Images were processed using Image J.

### Glucose-stimulated insulin secretion assay

5 × 10^5^ cells were seeded in 12-well plates and allowed to adhere for 24 h. Given the relatively high concentration of insulin in KSFM, cells were washed and cultured with CMRL-complete (CMRL 1066 supplemented with Antibiotic–Antimycotic [Gibco], and Glutamax [Gibco]) to reduce the background insulin concentration of the ELISA. Cells stimulated with EZH2 inhibitors or vehicle control (DMSO) for 2 or 7 days, following which they were washed with 2.8 mM glucose Krebs Buffer Solution (25 mM HEPES, 115 mM sodium chloride, 24 mM sodium hydrogen carbonate, 5 mM potassium chloride, 1 mM magnesium chloride heptahydrate, 0.1% bovine serum albumin, and 2.5 mM calcium chloride dihydrate made up in deionized water) two times, and incubated for 1 h to obtain the low glucose (basal) insulin secretion. Next, cells were cultured in 28 mM (High) glucose Krebs Buffer solution and incubated for 1 h to obtain the glucose stimulated insulin secretion. Supernatant was collected and Ultrasensitive Insulin ELISA (Mercodia) was used to determine the concentration of insulin according to manufacturer’s instructions. Fold change of insulin secreted by cells were calculated by adjusting to cells stimulated with EZH2 inhibitors for insulin concentrations compared to control.

## Supplementary Information

**Additional file 1.** Analysis of H3K9/14 acetylation content relative to total H3 shows no change following treatment with EZH2 inhibitors for 2- and 7-days.
