# Supplementary material for: Pharmacological inhibition of human EZH2 can influence a regenerative β-like cell capacity with in vitro insulin release in pancreatic ductal cells
Source: Clin Epigenetics. 2023 Jun 12;15:101. doi: 10.1186/s13148-023-01491-z (PMC10262441; doi:10.1186/s13148-023-01491-z)
Supplement: Supplementary file 1 — Additional file 1. Analysis of H3K9/14 acetylation content relative to total H3 shows no change following treatment with EZH2 inhibitors for 2- and 7-days. [file 13148_2023_1491_MOESM1_ESM.docx]

Supplementary Materials for

Pharmacological inhibition of human EZH2 can influence a regenerative β-cell like capacity with *in vitro* insulin release in pancreatic ductal cells

Safiya Naina Marikar^1,2^, Keith Al-Hasani^1,2^, Ishant Khurana^1,2^, Harikrishnan KN^1,2^, Jun Okabe^1,2^, Scott Maxwell^1,2^, Assam El-Osta^1,2,3,4,5,6^

^1^Department of Diabetes, Central Clinical School, Monash University, Melbourne, Victoria 3004, Australia, ^2^Epigenetics in Human Health and Disease Laboratory, Central Clinical School, Monash University, Melbourne, Victoria 3004, Australia, ^3^Department of Medicine and Therapeutics, The Chinese University of Hong Kong, Hong Kong SAR, ^4^Hong Kong Institute of Diabetes and Obesity, Prince of Wales Hospital, The Chinese University of Hong Kong, 3/F Lui Che Woo Clinical Sciences Building, 30-32 Ngan Shing Street, Sha Tin, Hong Kong SAR, ^5^Li Ka Shing Institute of Health Sciences, The Chinese University of Hong Kong, Hong Kong SAR, ^6^University College Copenhagen, Faculty of Health, Department of Technology, Biomedical Laboratory Science, Copenhagen, Denmark.

Corresponding author

Sam.el-osta@monash.edu

**This PDF file includes:**

Materials and Methods

Supplementary Figure 1

Table S1

Materials and Methods

**Protein Blot**

Histone proteins were extracted from 1x10^6 cells per sample. Acid extraction of nuclear proteins and immunoblotting was performed as previously described.^1^ Protein content of samples were incubated using Bradford’s Reagent (Sigma), with standard concentrations of BSA used to determine concentration. 1 ug of protein per sample was run on a 4-12% gel (Nu-Page, Invitrogen) before transfer to a PVDF membrane. Membranes (Immobilon-FL; Millipore) were incubated in primary antibody against H3K9/14ac (06-599, Millipore) or H3 (1B1B2, CST) overnight (dilutions listed in table 1). Membranes were incubated in secondary antibody and imaged using LiCoR Odyssey infrared system. Image studio was used to quantify the protein bands with total H3 as a loading control.


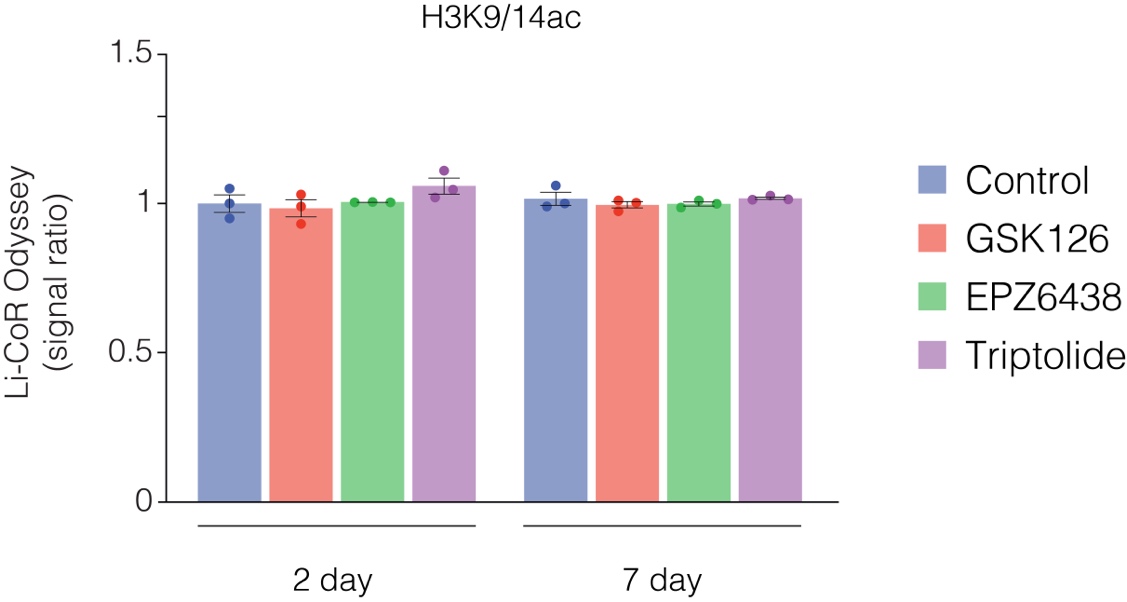


**Supplementary Figure 1.** Semi-quantitative analysis of H3K9/14ac relative to total H3 following 2-day and 7-day stimulation with GSK126 at 10 µM, EPZ6438 at 1 µM, Triptolide at 20 nM compared with vehicle control DMSO. Data are displayed as mean signal ratio of H3K9/14ac to total H3 ± SEM of 3 replicates. Statistically significant differences were determined using Student’s t-tests against control.

**Table S3**. Antibody dilutions for western blot human pancreatic ductal epithelial cells

| **Antibody** | **Dilution** |
| --- | --- |
| Total H3 | 1:1000 |
| H3K9/14ac | 1:1000 |
| IRDye® 680CW Goat Anti-Rabbit | 1:10000 |
| IRDye® 800CW Goat Anti-Mouse | 1:10000 |

**References**

1. Kaipananickal H, Waheed Khan A, Okabe J, Corcoran SJ, Esler MD, El-Osta A. Targeting Treatment Refractory NET by EZH2 Inhibition in Postural Tachycardia Syndrome. Circ Res. 2020;126(8):1058-60.
